# Enterovirus circulation in the WHO European region, 2015–2022: a comparison of data from WHO's three core poliovirus surveillance systems and the European Non-Polio Enterovirus Network (ENPEN)

**DOI:** 10.1016/j.lanepe.2025.101292

**Published:** 2025-04-11

**Authors:** Heli Harvala, Caroline K. Johannesen, Kimberley S.M. Benschop, Eugene V. Saxentoff, Shahin Huseynov, José E. Hagan, Thea K. Fischer

**Affiliations:** a Institute of Biomedicine, Medical Faculty, University of Turku, Finland; b Microbiology Services, National Health Service (NHS) Blood and Transplant, London, United Kingdom; c Department of Clinical Research, Nordsjællands Hospital, Hilleroed, Denmark; d Center for Infectious Disease Control, National Institute for Public Health and the Environment, Bilthoven, Netherlands; e Vaccine-preventable Diseases and Immunization, Division of Communicable Diseases, Environment and Health, World Health Organization (WHO) Regional Office for Europe, Copenhagen, Denmark; f Department of Public Health, University of Copenhagen, Copenhagen, Denmark

**Keywords:** Enterovirus, Paralysis, Surveillance, Typing, Severe, Europe, WHO, ENPEN

## Abstract

**Background:**

While the association of polioviruses with paralytic disease is well-documented and closely monitored via the Global Polio Eradication initiative, monitoring of the circulation and role of other non-polio enteroviruses in paralytic and non-paralytic disease has not received the same priority. We have assessed assess the role and potential effectiveness of the current enterovirus surveillance systems in the final stages of polio eradication.

**Methods:**

We compared data on enterovirus circulation and clinical associations reported to the World Health Organization (WHO) Regional Office for Europe via the acute flaccid paralysis (AFP), clinical enterovirus, and environmental surveillance systems along with that collected by the European Non-Polio Enterovirus Network (ENPEN), 2015–2022.

**Findings:**

This 8-year study analysed data from 63,659 samples from diagnosed enterovirus infections reported by 48 European countries, of which 27,699 were successfully typed (43.5%). This revealed the circulation of 67 individual enterovirus types primarily reported via ENPEN (85%; 19,712/23,220), whereas most poliovirus infections were reported via WHO (99.9%; 4484/4489). Only 20% of non-polio enterovirus positive AFP cases reported to WHO were successfully typed (105/544). Clinical data linked to these cases underscored the severity of paralytic non-polio enterovirus infections with 12 deaths compared to three deaths caused by poliovirus infections during the same study period.

**Interpretation:**

The study documents non-polio enterovirus infections as a frequent cause of paralysis in Europe. Implementation of standardized monitoring and reporting of all enteroviruses identified from severely ill patients, including those with paralysis, would enhance our understanding of the burden of non-polio enterovirus infections without compromising poliovirus surveillance.

**Funding:**

This study was funded by 10.13039/100004423WHO Regional Office for Europe and received financial support from the 10.13039/100000865Bill and Melinda Gates Foundation.

**Research in context**

**Evidence before this study**

Poliovirus was the first enterovirus recognised and linked to acute flaccid paralysis (AFP). Large epidemics of poliomyelitis in 1950s led to a development of two vaccines to protect against poliovirus infection and their inclusion in national childhood immunisation programmes. This measure was remarkably effective at preventing poliovirus-associated disease and interrupted the circulation of the virus in most parts of the world and prompted the establishment of the Global Polio Eradication Initiative programme in 1988. Gold standard surveillance in support of polio eradication focuses on investigating faecal samples collected from the individuals with acute flaccid paralysis by virus isolation using cell lines selective for poliovirus replication. Additional surveillance activities include testing of wastewater, and active clinical investigations of other clinical presentations potentially linked to poliovirus infections. Data from all three surveillance systems are reported to the World Health Organization (WHO) and used to inform public health actions. Although these surveillance systems are methodologically targeted towards poliovirus detection, it also reports data on non-polio enterovirus infections.

We searched PubMED for peer reviewed papers published in any language from inception to 21/10/2024 using terms “World Health Organization” AND enterovirus. This identified 25 publications, from which 19 focused on poliovirus, 5 described AFP surveillance in individual countries and the remaining were disease specific. To our knowledge, the surveillance data on non-polio enterovirus infections have not been previously systematically analysed and the extent of symptomatic infection with non-polio enteroviruses remains unreported.

The European Non-Polio Enterovirus Network (ENPEN) was established to improve the awareness, diagnosis and available data on non-polio enterovirus infections in Europe. While the initial work has already provided evidence that non-polio enteroviruses often cause sever neurological infections, their full disease burden has remained undefined.

**Added value of this study**

For the first time, this study has combined the typing and clinical data on enterovirus infections reported to the ENPEN with the data reported to the three WHO poliovirus surveillance systems by 48 countries in the Europe between 2015 and 2022. A vast dataset of 63,659 enterovirus-positive samples were included in this study; from these, typing of 27,699 revealed the circulation of 67 enterovirus types. The study demonstrated further that while most poliovirus data was indeed reported to the WHO (99.9%; 4484/4489), ENPEN captured a huge additional amount of largely unreported data on non-polio enterovirus infections (85%; 19,742/23,220). The study findings document the often-severe nature of non-polio enterovirus infection, especially enterovirus D68 and A71 that were often associated with paralysis, including 12 fatalities; these greatly exceed disease metrics of poliovirus infections over the same period.

**Implications of all the available evidence**

Our study highlights the role of numerous non-polio enteroviruses as a cause of paralysis and other severe neurological disease. These findings advance the case for more systematic investigation of patients presenting with neurological infections that may be linked to enterovirus infections. We propose that all patients with severe neurological symptoms including paralysis, acute flaccid myelitis and meningitis should be screened for enteroviruses utilising appropriate molecular methods and correct sample types, and enterovirus types identified in the positives. This will not only help better define the burden of non-polio enterovirus infections, but also support and extend existing poliovirus surveillance activities. Alignment of ENPEN surveillance data collection with the WHO framework will enable far more detailed monitoring of disease activities of all enteroviruses.

## Introduction

Members of the four species of the human *Enterovirus* genus comprise of over 100 non-polio enterovirus and three poliovirus types. While the association of polioviruses with paralytic poliomyelitis is well known, other enteroviruses display diverse clinical associations that include paralysis, neurological and respiratory infections to hand, mouth, and food disease (HMFD) and occasionally very severe neonatal infections. While many enterovirus infections are asymptomatic, frequencies of the most severe outcomes are largely unknown limiting the estimation of their true disease burden. For these reasons, it is important to identify enterovirus types associated with more severe conditions. Virus characterization is also important for the confirmation of the absence of circulation and importation of polioviruses, and for monitoring the (re)-emergence of novel enterovirus types.

Poliovirus was the first enterovirus to be recognised, subsequently linked to acute flaccid paralysis (AFP). Epidemics of poliomyelitis in the 1950s motivated the urgent development of oral and inactivated poliovirus vaccines (OPV/IPV), and the implementation of universal childhood immunisation, and the subsequent establishment of the Global Polio Eradication Initiative programme in 1988. Through these efforts, the circulation of wild type polioviruses (WPV) ceased worldwide, apart from small endemic foci of WPV1 in parts of Afghanistan and Pakistan.^1^ However, numerous and often paralysis-associated vaccine-derived poliovirus (VDPV) infections are recorded worldwide, including in Europe, underlining the necessity to maintain strong population immunity through universal vaccination and ongoing extensive surveillance for poliovirus.^2^, ^3^, ^4^, ^5^, ^6^

The ultimate aim of poliovirus eradication is to stop its circulation in the population. For this objective, it has been vital to have a surveillance strategy to monitor the presence of polioviruses in faecal specimens of AFP cases, where the virus is detectable in high quantities and for long periods after infection (typically 3–6 weeks).^7^ However, many countries have chosen to implement alternative or additional surveillance systems for polioviruses. These supplemental surveillance approaches include environmental testing, such as wastewater, and active clinical investigations of disease presentations potentially linked to poliovirus and other enterovirus infections. Clinical surveillance focuses on the detection of polioviruses in samples collected from patients with symptoms of suspected poliovirus, or patients testing positive for enterovirus, to confirm that poliovirus is not causing clinical disease. In environmental surveillance, wastewater samples are systematically collected and screened for poliovirus by virus isolation in selective cell lines.^8^ It can demonstrate the circulation of poliovirus and other enterovirus types independently of their clinical presentations.^9^^,^^10^

The European Non-Polio Enterovirus Network (ENPEN) was established in 2017 under the auspices of the European Society for Clinical Virology as a supra-national, non-commercial, core reference consortium.^11^ By bringing together interdisciplinary specialists from over 30 European countries, it aims to raise professional awareness for more effective detection, develop diagnostic and typing tools, guide surveillance activities and enhance public health response.

In this study, we have compared data that was retrospectively collected from participating institutes within the ENPEN network between 2015 and 2022 on enterovirus circulation and clinical associations in Europe where poliovirus has largely been eradicated^12^ with the data provided by the Member States to WHO over the same time period. The study contrasts the extent of typing and clinical EV data reported through the ENPEN surveillance with data from the same countries through AFP, clinical enterovirus and environmental surveillance systems collected by WHO. The study also evaluates the role and potential effectiveness of each surveillance system in the final stages of polio eradication.

## Methods

The WHO European region covers 53 countries with a total population of ∼929 million in 2021.^13^ The countries, their use of surveillance systems and strategies are described in Table 1. Country-specific surveillance data and the number of samples subjected to enterovirus testing reported by the countries included in this study was collected between 2015 and 2022. Aggregated ENPEN surveillance data was retrospectively collected from 2015–2017 to 2018–2022.

**Table 1:** Surveillance systems, number of samples subjected to enterovirus testing and reported and population size for countries in the WHO Europe, 2015–2022.

Country	Population	Number of samples reported to AFP surveillance	Number of samples reported to Environmental surveillance (% of population covered)	Number of samples reported to Clinical Enterovirus surveillance (% of population covered)	ENPEN network
Albania	2,832,439	60	**No**	640	No
Andorra	79,034	0	**No**	**No**	No
Armenia	2,777,971	201	**No**	0 (100)	No
Austria	8,958,960	80	**No**	0 (99)	Yes
Azerbaijan	10,412,652	337	2369 (20)	**No (reported positives)∗**	No
Belarus	9,498,238	932	79 (88)	24 (100)	No
Belgium	11,594,060	0	**No**	0 (100)	Yes^b^
Bosnia Herzegovina	3,210,847	66	**No**	**No**	No
Bulgaria	6,687,717	180	**No**	0	Yes
Croatia	4,008,61	20	2245	1782	No
Cyprus	1,260,138	16	**No**	0	No
Czech Republic	10,495,295	113	1387	**No (reported 308)∗**	Yes^a^
Denmark	5,964,059	**No**	**No**	4498 (100)	Yes
Estonia	1,322,765	12	449 (49)	943 (100)	Yes^a^
Finland	5,545,475	**No (reported 63)∗**	574 (29)	**No (reported 86)∗**	Yes
France	67,750,000	**No**	**No**	2966 (99)	Yes^b^
Georgia	3,728,282	142	722 (47)	ND (reported positives)	No
Germany	83,200,000	**No**	162 (2)	1491 (100)	Yes
Greece	10,341,277	207	88	466	No
Hungary	10,156,239	165	**No**	**No**	Yes^a^
Iceland	37,252	**No**	**No**	0 (100)	Yes^a^
Ireland	5,056,935	2	**No**	218 (100)	No
Israel	9,496,000	369	2010 (63)	46	No
Italy	58,870,762	619	1756 (11)	**No (reported 15)∗**	Yes
Kazakhstan	19,606,634	2317	52 (100)	6 (100)	No
Kyrgyzstan	6,735,348	923	**No**	**No**	No
Latvia	1,830,211	68	747 (65)	6 (100)	Yes^a^
Lithuania	2,718,352	119	**No**	1571 (100)	Yes^a^
Luxemborg	672,051	**No**	**No**	**No**	Yes^a^
Malta	535,064	55	1 (100)	**No**	No
Moldova	3,435,931	2	9 (12)	0 (28)	No
Monaco	36,686	0	0	**No (reported 1889)∗**	No
Montenegro	626,485	16	**No**	**No**	No
Netherlands	17,530,000	**No**	1192 (6)	76 (100)	Yes
North Macedonia	2,085,679	0	**No**	**No**	No
Norway	5,474,360	219	**No**	0 (100)	Yes
Poland	41,026,067	645	137 (6)	**No (reported 713)∗**	Yes^a^
Portugal	10,247,605	54	**No**	7 (100)	Yes^a^
Romania	19,892,812	533	1839 (34)	**No (reported 7)∗**	Yes^a^
Russian Federation	144,444,359	6995	6052 (100)	5280 (100)	No
San Marino	33,745	**No**	**No**	**No**	No
Serbia	7,149,077	158	**No**	**No (reported 187)∗**	No
Slovakia	5,795,199	38	2139 (54)	6503 (100)	Yes^a^
Slovenia	2,119,675	27	**No (reported 7)∗**	1788 (100)	Yes
Spain	47,519,628	463	7	57 (7)	Yes^a^
Sweden	10,467,097	**No**	**No**	417 (100)	Yes
Switzerland	8,796,669	79	**No**	**No**	Yes^b^
Tajikistan	10,078,507	2223	26 (17)	**No**	No
Turkmenistan	85,816,199	4883	**No (reported 54)∗**	**No (reported 97)∗**	No
Türkiye	6,516,100	554	**No**	ND (reported positives)	No
Ukraine	36,744,634	5864	423 (100)	606 (100)	No
United Kingdom	67,736,802	90	368 (34)	497 (100)	Yes
Uzbekistan	34,739,400	2077	540 (7)	**No**	No

No indicates countries that do not conduct related surveillance (also marked in bold) and those reporting despite not formally participating to the surveillance marked with∗.

a Countries reporting to ENPEN in 2015–17 only.

b Countries reporting to ENPEN in 2018–2022 only.

### Enterovirus data reported to WHO

Case-level data was collected through Online Laboratory Data Management System (LDMS) and the Centralised Information System for Infectious Diseases (CISID). Although these surveillance systems are methodologically targeted towards poliovirus detection, it also reports data on non-polio enterovirus infections in individuals presenting with polio-like symptoms. Information on the collection date, country, and province of sample origin were provided for all samples. The number of samples tested and enterovirus-positives as well as available typing results were reported.

These three types of surveillance systems were included:

1) AFP surveillance conducted by 44 countries: Case-based syndromic surveillance for AFP cases by testing specimens primarily from cases and contacts to cases.^14^ Reported sample types were cerebrospinal fluid (CSF), faecal, respiratory, and unknown/other. In AFP surveillance, further data on clinical symptoms and outcome are carefully reviewed to confirm whether cases full-fill the case definition.
2) Clinical enterovirus surveillance conducted by 32 countries, and 26 from these at national level: Samples were from cases of suspected polio or their contacts, or other illness with symptoms of enterovirus infections.^15^ Sample types were CSF, faecal, respiratory, and unknown/other. While some countries report all samples, others report only samples testing positive for enterovirus, some focus on poliovirus-positive samples only, or some on samples subjected to poliovirus testing.
3) Environmental surveillance conducted by 26 countries: Wastewater and other environmental samples were systematically collected and tested for poliovirus in specific settings.^9^

### Clinical surveillance data on enterovirus collected by ENPEN

ENPEN collects data on enteroviruses reported via the existing national (or local) surveillance systems in the EU/EEA region. Aggregated information includes sample type, age group, typing results, and other locally relevant information and is submitted via the national laboratory, a network of laboratories or by individual hospitals. The data used in this paper was collected in two cycles, one covering 2015–2017,^12^ and one covering 2018–2022 (Sten et al., manuscript)^16^ from a total of 25 countries (Tables 1 and 2). Sample types were biopsy, blood/serum, CSF, faecal, respiratory, vesicle, environmental, and unknown/other. Data on age, clinical and outcomes were also obtained in aggregated format.

**Table 2:** Total number of samples reported, and samples tested positive for enterovirus per reporting country and WHO surveillance system in the WHO Europe region 2015–2022 (no EV-positive AFP cases reported by Macedonia [not shown]).

	AFP	NPEV typed	Typed PV	Typed (%)^a^	Clinical enterovirus				Environmental				ENPEN	Typed NPEV	Typed (%)
	EV-positive				EV-positive	Typed NPEV	Typed PV	Typed (%)^a^	EV-positive	Typed NPEV	Typed PV	Typed (%)^a^	EV-positive		
Total	1737	388	622	58	11,039	1409	394	16	8091	1677	3453	64	44,555	19,712	44.2
Albania	2	0	2	100	39	0	0	0		0	0				
Armenia	18	0	10	56		0	0			0	0				
Austria	4	4	0	100		0	0			0	0		473	410	86.7
Azerbaijan	10	4	5	90	19	18	0	95	71	37	22	83			
Belgium	0	0	0		0	0	0		0	0	0		2680^c^	1623	60.6
Belarus	16	1	4	31	6	0	6		40	0	40	100			
Bosnia-Herzegovina	4		4	100											
Bulgaria	6	4	0	67		0	0			0	0		175	46	26.3
Croatia	5	5	0	100	170	134	0	79	9	6	0	67			
Cyprus	2	0	0	0		0	0			0	0				
Czech Republic	9	0	0	0	77	21	0	27	310	6	1	2	118^b^	118	100
Denmark		0	0		4497	0	0	0		0	0		3866	2164	56.0
Estonia	0	0	0		56	42	0	75	139	127	0	91	27^b^	12	44.4
Finland	0	0	0		12	11	0	92	522	20	20	8	1195	134	11.2
France		0	0		2965	0	0			0	0		10,511^c^	7482	71.2
Georgia	15	0	8	53	41	2	1	7	205	3	41	19			
Germany		0	0		325	0	1		153	75	24	65	2361	1066	45.2
Greece	11	9	0	82	72	48	0	67	36	19	0	53			
Hungary	1	1	0	100		0	0			0	0		112^b^	105	93.8
Iceland	0	0	0		0	0	0		0	0	0		103^b^	79	76.7
Ireland	0	0	0		178	107	0	60		0	0				
Israel	25	19	2	84	33	0	33		1556	0	1536	99			
Italy	14	10	0	71	6	1	0	17	615	131	3	22	812	202	24.9
Kazakhstan	110	35	24	54	3	0	3		29	5	25	103			
Kyrgyzstan	92	28	27	60		0	0			0	0				
Latvia	4	3	0	75	1	1	0	100	211	164	0	78	104^b^	16	15.4
Lithuania	14	12	0	86	26	13	3	62		0	0		22^b^	22	100
Luxemborg		0	0		0	0	0		0	0	0		231^b^	6	2.6
Malta		0	0			0	0		1	0	0	0			
Mvaoldo	6	0	5	83	96	28	8	38	382	128	137	69			
Montenegro	1	0	0	0	0	0	0			0	0				
Netherlands		0	0		2	0	2		625	561	16	92	3885	1751	45.1
Norway	13	12	0	92		0	0			0	0		882	519	58.8
Poland	21	13	0	62	244	27	0	11	120	0	23	27	137^b^	137	100
Portugal	5	0	1	20	3	0	3			0	0		27^b^	27	100
Romania	39	0	0	0	2	0	0	0	574	0	0	0	9^b^	9	100
Russia	303	48	158	68	983	404	198	61	1414	205	1137	95			
Serbia	4	2	0	50	27	9	0	33	0	0	0				
Slovakia	3	0	0	0	250	145	1	58	265	171	6	67	130^b^	96	73.8
Slovenia	1	0	0	0	345	117	0	34	1	1	0	100	876	397	45.3
Spain	14	12	1	93	31	21	7	90		0	0		5215	1799	34.5
Sweden		0	0		211	193	0	91		0	0		2061	524	25.4
Switzerland	1	0	0	0		0	0			0	0		257^c^	0	0.0
Tajikistan	340	68	140	61		0	0		75	4	67	85			
Turkey	234	49	33	35	9	6	3	100	0	0	0				
Turkmenistan	75	19	6	33		0	0			0	0				
Ukraine	222	27	177	91	194	53	113	86	214	13	178	89			
United Kingdom	4	0	0	0	116	8	12	17	348	1	162	47	7785	968	12.4
Uzbekistan	89	3	15	20		0	0		166	0	16	10			

NPEV, non-polio enterovirus; PV, poliovirus, could be wild or vaccine-derived poliovirus.

a Typing rate for PV typed and NPEV typed combined.

b Countries reporting to ENPEN in 2015–17 only.

c Countries reporting to ENPEN in 2018–2022 only.

### Ethics

As only anonymous surveillance data was collected in this study, specific ethical approval was not needed.

### Missing data and statistical analysis

WHO Europe provided information on samples reported for AFP surveillance, clinical enterovirus surveillance and environmental surveillance, and ENPEN on data already collected. All enterovirus-positive samples were included in the analysis. However, it was not possible to differentiate between samples taken from the same case or location. Consequently, multiple samples from the same case or site may be represented in the results. Furthermore, no data was collected on laboratory methods used for the detection or typing of enteroviruses. However, in general, most countries would follow the WHO guidance when participating to AFP, environmental or clinical surveillance.^9^^,^^14^^,^^15^ These focus on the exclusion of poliovirus and hence are optimised towards the poliovirus detection, relying on virus isolation followed by typing. Countries reporting data via ENPEN would most likely apply molecular-based methods according to the recent recommendations.^11^

With optional reporting of non-polio enterovirus to the WHO surveillance systems and network-based reporting of enterovirus types to the ENPEN, missing data is expected, and no assumption of randomness can be applied. For the ENPEN data 2018–2022 specifically, the missing data is non-random, as the participating partners report only the top ten enteroviruses typed. Both testing and typing are also likely biased towards the more severe clinical presentations. The missing data contributes to the underestimation of circulation of enteroviruses in Europe. We performed aggregation on enterovirus type, country and surveillance system and computed totals, subtotals and proportions. Data management and analyses were conducted using R studio version 2023.9.0.463.^17^^,^^18^

### Role of the funding source

This work was funded by the WHO regional office for Europe. The data was provided by the funding source. The work was conducted by the ENPEN study group in close collaboration, and input on the study design and the drafted manuscript allowed.

## Results

During the 8-year study period, 49 countries of the WHO European Region reported enterovirus data for at least one of the surveillance systems (Tables 1 and 2). Of these countries, 45 reported to the AFP surveillance data, 41 contributed to clinical surveillance and 28 reported data on environmental surveillance. ENPEN surveillance data was obtained from 26 countries. All countries except four small countries reported data either to WHO or ENPEN during the study period (Table 1). Data collected via the ENPEN surveillance was national for all except one country (Italy).

### Numbers of samples tested reported

A total of 89,150 samples subjected to enterovirus (including poliovirus) testing were reported to WHO with an average of 11,143 samples per year (Tables 3 and 4); AFP: 31,071 samples (34.9% of all samples), clinical enterovirus surveillance: 32,459 (36.4%), and environmental surveillance: 25,620 (28.7%). A total of 539,792 samples subjected to enterovirus testing had been reported through ENPEN surveillance.

**Table 3:** Countries reporting, samples collected and tested for enteroviruses, as well as samples found to be positive for enterovirus per year and surveillance system.

	Year	2015	2016	2017	2018	2019	2020	2021	2022	Total
AFP	Countries reporting	36	39	41	39	38	34	33	33	
	Total	4018	4794	4439	4162	4140	2356	3382	3780	31,071
	EV	297	258	245	249	234	65	254	135	1737
	EV Types identified	28	25	22	22	15	3	8	9	
Clinical Enterovirus	Countries reporting	24	26	26	29	29	22	24	23	
	Total	2548	4488	3951	4025	4421	1893	4868	6365	32,559
	EV	640	847	1070	1176	1476	340	1767	3723	11,039
	EV Types identified	28	33	27	32	25	10	14	24	
Environmental	Countries reporting	18	19	20	24	24	19	21	20	
	Total	2495	3470	3332	3274	3172	2527	3440	3910	25,620
	EV	769	816	1027	1061	1051	594	941	1832	8091
	EV Types identified	31	19	24	25	28	20	30	26	
ENPEN	Countries reporting	24			15	15	15	16	16	
	Total	21,086			96,936	101,863	113,780	46,387	159,740	539,792
	EV	4144	6084	5686	7769	6836	2089	3681	8266	44,555
	EV Types identified	52	51	45	28	29	31	27	25

**Table 4:** Sample types, all and positive for non-polio enterovirus, by surveillance system, number of samples N (% of total), positive (% of total) and P% (positive percentage), noting only samples where sample type was reported are included.

Sample type	AFP (%)			Clinical enterovirus (%)			Environmental (%)			ENPEN^a^ (%)	
	N	Positive	P%	N	Positive	P%	Samples	Positive	P%	N	Positive
Biopsy											2 (0.04)
Blood/Serum											329 (6.71)
CSF	69 (0.22)	3 (0.24)	4.35%	3802 (12.38)	1488 (17.42)	39.14%					1054 (21.51)
Faecal	31,834 (99.51)	1261 (99.37)	3.96%	23,195 (75.56)	4274 (50.05)	18.43%					1673 (34.14)
Respiratory	88 (0.28)	5 (0.39)	5.68%	3702 (12.06)	2778 (32.53)	75.0					1597 (32.59)
Vesicle											246 (5.02)
Environmental							25,855 (100)	4197 (100)	16.23%		

a No information on EV-negative samples was collected via the ENPEN study. Sample type only available for years 2018–2022.

### Numbers of EV-positive samples reported

While yearly numbers of reported and positive samples fluctuated along with the number of countries reporting for each surveillance system, a total of 20,867 enterovirus-positive samples from all three systems were reported to WHO over the study period (average of 2608 EV-positive samples per year; Tables 3 and 4). However, positive reporting rates varied greatly between sample types and surveillance methods; AFP surveillance contributed 1737 enterovirus-positive samples (5.6%) whereas 11,039 positives were reported via the WHO clinical enterovirus surveillance (52.9%) and 8091 positives originated from the environmental surveillance (38.8%). A total of 44,555 enterovirus positives samples (8.3% of 539,792) were reported by clinical ENPEN surveillance in the study period (average 5569 positive samples per year).

### Sample types

Most reported sample type tested came from cases (n = 50,083; 56.2%), contacts (n = 12,873; 14.4%) and environmental samples (n = 25,859; 29.0%). AFP surveillance primarily reported testing of faecal samples (30,856; 99.3%), of which 3.5% were positive for non-polio enteroviruses (Tables 3 and 4). In clinical enterovirus surveillance, the most reported sample type tested was faeces (22,082, 68%), whereas respiratory samples were more often positive for non-polio enterovirus than faecal samples (2778/3692, 75% versus 4100/22,082, 19%). Furthermore, similar numbers of non-polio enterovirus positives reported via the ENPEN surveillance originated from CSF, faecal and respiratory samples (1054, 10%, 1673; 16%, 1597; 15% respectively, while sample type was not known for 5573 samples).

### Enterovirus-positive samples successfully typed

Of the 1737 positive samples reported via AFP surveillance, 388 were successfully typed as non-polio enterovirus and 632 were identified as poliovirus (Table 2, Table 3, Table 4). In clinical surveillance, of the 11,039 positive samples, 1409 (12.7%) samples were successfully typed as non-polio enterovirus and 394 (3.6%) as polioviruses, whereas from 8901 positives identified via the environmental surveillance, 1681 (18.8%) were typed as non-polio enterovirus and 3453 (38.8%) as polioviruses. In the ENPEN surveillance, of the 44,555 positives, 19,712 (44.2%) were successfully typed as non-polio enterovirus and five as poliovirus (<0.01%).^19^

### Typing and clinical data reported to WHO and the ENPEN network

**Fig. 1:**
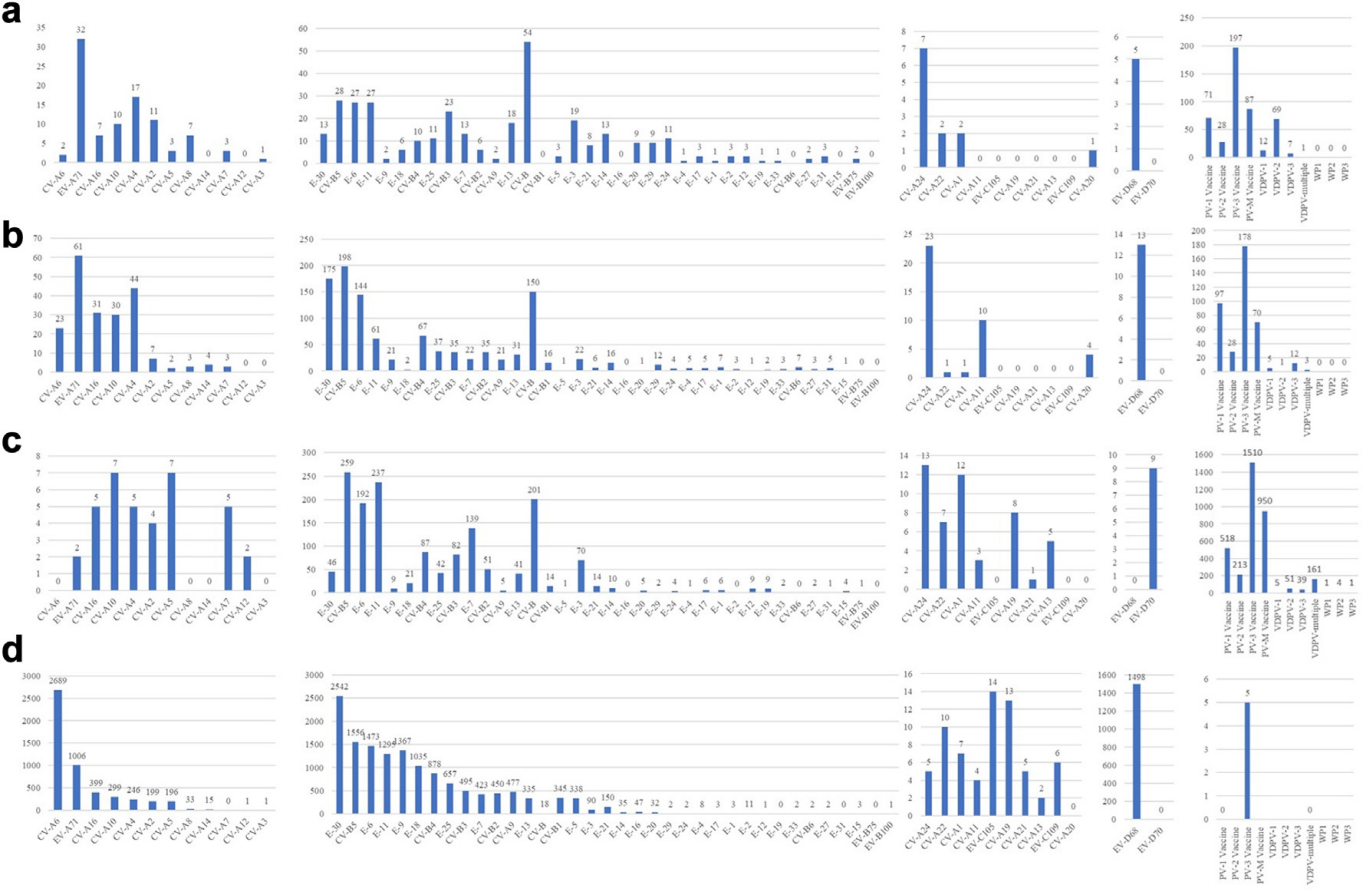
Number of enterovirus types divided into species a, b, c and d identified by the following surveillance systems a) AFP surveillance, b) clinical enterovirus surveillance, c) environmental surveillance, and d) ENPEN surveillance in Europe, 2015–2022. Note changing y-axis in all graphs. PV-M: samples with detection of multiple PV-types.

During the study period, 67 non-polio enterovirus types were detected, including 13 from species A, 40 from species B, 12 from species C and 2 from species D (Fig. 1). The number of types reported by year varied in each surveillance and was lowest during the COVID-19 pandemic in 2020–2022 (Tables 3 and 4; Supplementary Figure S1).

Further comparison of enterovirus types identified through the ENPEN surveillance with those reported by the same countries through the AFP, clinical and environmental surveillance systems was based on 23,220 positive samples reported by 25 European countries (Fig. 1). Most data originated from the ENPEN surveillance (85%; 19,712/23,220). The 10 most reported types were E30 (n = 2776, 12%), CVA6 (n = 2714, 11.7%), CVB5 (n = 2041, 8.8%), E6 (n = 1836, 7.9%), E11 (n = 1620, 6.9%), EV-D68 (n = 1516, 6.5%), E9 (n = 1399, 6%), EV-A71 (n = 1101, 4.7%), E18 (n = 1064, 4.6%) and CVB4 (n = 1042, 4.5%). Some specific types known to cause severe disease, such as EV-D68 and CVA6, were not found in the environmental surveillance and were only rarely reported via the AFP and clinical surveillance (5 and 13 cases of EV-D68, respectively; 2 and 23 cases of CVA6). EV-D70 was found only once via environmental surveillance, and CVA7 was reported via all three WHO surveillance systems. Almost all polioviruses were identified and reported via the WHO surveillance systems (341/346; 98%). These included vaccine strains of poliovirus (n = 251), VDPV (n = 84) and WPV (n = 6; further details in Fernandez-Garcia et al., 2021).^19^ Furthermore, 5 VDPV-positive samples were reported via the ENPEN surveillance although not presented in previous ENPEN paper as they were not among the top 10 types reported.^19^

Interestingly, 79 cases of acute flaccid myelitis (AFM) were reported by 12 countries during the first ENPEN study in 2015–2017 and 71 of these were successfully typed.^12^ The most frequently reported NPEV types in these patients were EV-D68 (n = 31) and EV-A71 (n = 24). In comparison, during the same period, 262 non-polio enterovirus cases were reported to the AFP surveillance and 67 of these successfully typed. The three most common types were EV-A71 (n = 21), CVB5 (n = 7) and EV-D68 (n = 13), reported by 7 countries (Supplementary Figure S2). The detailed nature of clinical data collected by WHO enabled further comparison of poliovirus vaccine related cases with those associated with non-polio enteroviruses (Table 5). Similar mortality was reported for poliovirus vaccine-related cases and non-poliovirus cases (1.3%, 3/233 versus 2.2%, 12/544). Most reported cases where non-polio enterovirus type had been identified were young children and presented with limb paralysis, which in 20% cases was polio-like. Around 39% of cases had residual weakness (highest for EV-D68: 72%) and around 3% of reported cases had died (highest for EV-A71: 10%).

**Table 5:** Reporting characteristics for the vaccine related poliovirus (PV) cases (n = 233) or non-polio enterovirus (NPEV) cases (n = 544) to the WHO AFP surveillance in the WHO Europe region 2015–2022.

Acute flaccid paralysis (AFP) surveillance	Detection of vaccine related PV	Vaccine-associated paralytic polio (VAPP)	Vaccine-derived polio (VDPV)	Detection of NPEV	Detection of NPEV with typing	Detection of EV-A71	Detection of EV-D68
Number of reported cases	233	24	41	544	105	21	13
Proportion of males	64.8%	79.2%	63.4%	56.4%	59.0%	52.4%	69.2%
Age distribution							
below 1 year	45	10	9	33	8	6	1
1–2 years	95	9	18	101	24	6	2
2–3 years	38	3	3	117	26	4	2
3–5 years	24	1	9	146	17	2	2
5–10 years	21	0	2	100	21	2	5
over 10 years	10	1	0	47	9	1	1
Reported symptoms							
Paralysis involving limbs	229	24	40	514	102	21	13
Additional respiratory paralysis	2	1	0	21	8	3	3
Asymmetric paralysis	124	14	18	158	40	10	8
Symmetric paralysis	105	10	23	378	62	9	5
Fever	136	22	35	223	54	18	7
No fever	96	2	6	306	57	3	5
Outcome							
Residual weakness	91	22	33	144	37	4	8
No residual weakenss	126	2	2	311	58	13	3
Death	3	0	1	12	3	2	0
Lost to follow-up/No data	13	0	5	77	7	2	2
Final clinical diagnosis							
GBS	75	1	31	321	43	4	2
Peripheral neuropathy	6	0	0	30	12	3	1
Polio-like	1	20	6	21	9	2	4
Transverse myelitis	7	0	0	38	10	2	5
Other/Unknown	78	3	1	134	31	9	1
Prior poliovirus vaccine							
Inactivated poliovirus vaccine	52	0	2	178	55	14	11

All AFP cases associated with vaccine related PV were further reviewed: clinical diagnosis of acute flaccid paralysis (AFP) was confirmed, and vaccine related poliovirus classified either as vaccine-associated (cause of vaccine-associated paralytic polio, VAPP) or vaccine-derived poliovirus (VDPV). Cases associated with NPEV were discarded from the surveillance focusing on polio, and hence their clinical details were not reviewed. EV-A71, enterovirus A71; EV-D68, enterovirus D68.

## Discussion

We have compared data reported to the AFP surveillance, clinical surveillance, and environmental surveillance along with the ENPEN surveillance in Europe from 2015 to 2022. The study provides the most extensive analysis of enterovirus circulation to date, reporting 63,659 enterovirus-positive samples of which 27,699 were successfully typed (43.5%). Most non-polio enterovirus typing data was reported via the ENPEN (85%; 19,712/23,220) whereas most polioviruses were reported via the WHO surveillance systems (99.9%; 4485/4490), reflecting the differences in their focus, sample types and detection methods. The large amount of typing and associated clinical data provided the opportunity to meaningfully compare the neurological and systemic disease burden and mortality of polioviruses with those of other enteroviruses. This study demonstrates that non-polio enteroviruses were a far more frequent cause of paralysis and other neurological disease than polioviruses in Europe.

The surveillance data shows the diverse nature of the WHO European Region in terms of EV infections and related surveillance. Whereas non-polio enterovirus surveillance is largely based on molecular detection of viral RNA directly in clinical specimens, poliovirus surveillance still relies on initial virus isolation in selective cell lines. A remarkable 4485 poliovirus-positive samples were reported during the study period, primarily through reporting to WHO. Although most of these viruses were identified from environmental samples as vaccine strains, relating to the recent use of OPV in the countries where they were detected, significant findings included the identification of 66 AFP cases linked to vaccine-related or vaccine-derived polioviruses, and 6 samples with WPV that originated from vaccine production facility containment breach incidents highlighting the value of environmental monitoring of poliovirus essential facilities.^20^ The marked contrast in detection rates of polioviruses and non-polio enteroviruses between countries primarily reflects differences in vaccine use; countries where OPV is still used in their vaccination program or for outbreak control, require a focus on poliovirus surveillance, including the use of selective testing methods at the expense of non-polio enterovirus detection. Contrastingly, non-polio enterovirus data is starting to emerge primarily from countries within the ENPEN network. Although it is largely based around testing of cases admitted to hospital with potential enterovirus infections, data collection is yet to be harmonized between countries.

Globally, there is an increasing focus on non-polio enterovirus surveillance due to the severity of emerging non-polio enterovirus infections.^21^ Although this and our previous study highlight the status of poliovirus-focused surveillance in Europe,^22^ the same issues are applicable elsewhere. The Asia–Pacific Network for Enterovirus Surveillance (APNES) focuses on development of standardized protocols and countries like Japan, China and South Korea have robust surveillance systems in place, often integrating non-polio enterovirus surveillance with other virus surveillance programs.^23^ The United States Center for Disease Control and Prevention conducts non-polio enterovirus surveillance through the National Enterovirus Surveillance System (NESS) and collects data from laboratories across the country as well as collaborates with states health departments to monitor outbreaks.^24^^,^^25^ In countries within the Africa, Middle East and Latin America surveillance efforts vary greatly; Brazil and South Africa have well-established enterovirus surveillance systems, and Israel has rather advanced wastewater surveillance for enteroviruses, whereas most other countries in these regions often focus on poliovirus eradication efforts and responding to outbreaks.^26^ In countries with surveillance and virus typing in place, new emerging non-polio enterovirus types such as those reported in our study, are detected.^27^ These viruses are rarely confided to one continent but presenting with a global circulation pattern.

The comparability of data acquired via the WHO and voluntary participation in ENPEN program is limited by the lack of harmonized case definitions as well as differences in testing and data reporting. However, data collected via the ENPEN provided important insights into the circulation, nature and clinical impact of enterovirus infections, including age groups affected and specific clinical syndromes associated with certain types.^12^ For example, it showed that disease presentations in around 40% of reported non-polio enterovirus infections were neurological in nature and underlines the association of many types, such as EV-D68 and EV-A71, with AFM or other neurological presentations. The 2015–2017 data also highlighted that CVA6 had become the most common type in Europe, with recent modelling indicating that this resulted from a major change in its transmissibility,^28^ and the same has been subsequently proposed for EV-D68.^29^ The extent of circulation of these and other non-polio enterovirus types in Europe and the associated clinical picture reported by ENPEN shows a great added value to WHO data as only a small number of EV-D68, EV-A71 and CVA6 positive samples were reported to the WHO during the 8-year study period (n = 18, 95 and 24, respectively). While enteroviruses are small RNA viruses, they are prone to genetic change and recombination; this might lead to change their transmissibility and pathogenesis and should be considered as another important reason to enhance clinical non-polio enterovirus surveillance.

Although polioviruses were the major enterovirus types linked to AFP for some decades, other non-polio enterovirus types are increasingly recognized and associated with a similar paralytic illness. Based on the AFP surveillance data analysed in this study, 46 different non-polio enterovirus types were identified in 544 samples obtained from AFP cases and their contacts. The most commonly identified types were EV-A71 and EV-D68; a finding which has been replicated in recent ENPEN and other studies.^12^^,^^21^ Further individual clinical data reported to the WHO on AFP cases clearly highlighted the severity of paralytic non-polio enterovirus infections; with 12 deaths compared to only three among those infected with VDPVs (Table 5). This rate is similar, if not higher, than that of paralytic poliomyelitis (2–10%). Furthermore, the risk of residual post-infectious weakness in non-polio enterovirus cases (particularly by EV-D68) should also be noted. These data provide evidence for a substantive role of many non-polio enteroviruses beyond polioviruses with paralytic illness and emphasize the importance of broadening the focus of surveillance to cover all enterovirus types. In parts of Europe where OPV is no longer used, WHO surveillance is still largely focused on polioviruses even though there were four times more non-polio enterovirus associated deaths reported via this surveillance than by polioviruses. While the newly established hospital based ENPEN surveillance will help to capture the true burden of severe enterovirus infections,^22^, ^30^ modifications into the current AFP surveillance should also be considered to allow this data to be collected.

The selectivity of AFP surveillance for polioviruses originates from isolation methods using RD and L20b cells and a poliovirus-specific PCR for type identification in cultures with cytopathology. The reliance on faecal samples for surveillance furthermore precludes identification of many non-polio enterovirus types, such as EV-D68 that are primarily excreted in respiratory samples.^11^ Collection of additional sample types, expansion of the case definition to include AFM and application of molecular detection followed by typing of all AFP cases should be urgently considered. During this study period, only 35% of non-polio enterovirus positive samples obtained from AFP cases were subjected to typing and/or successfully typed, illustrative of the degree of underlying by this surveillance (388/1105, Table 2). Data reported to WHO on AFP cases demonstrated this even further with only 20% of non-polio enterovirus cases successfully typed (105/405, Table 5). Without further investigations, we cannot state if the low typing frequency reflects a primary focus on only identifying poliovirus infections, poor performance of typing methods or potentially a lack of reporting. However, with the modern technology and small sample numbers, typing of most non-polio enterovirus positive AFP cases should be achievable and made a public health priority in the post-polio world.

Surveillance data should inform public health measures. A case of polio leads to several urgent public health actions including vaccinations, and further enhanced surveillance to monitor the effectiveness of control measures; this is based on our understanding that one case of polio would usually translate to 100 to 200 other subclinical poliovirus infections which have been missed. An equivalent attention on non-polio enteroviruses known to cause severe disease in alerting public health authorities and the local health care system should also be considered. Although environmental surveillance can be used to support polio eradication and to monitor changes in the epidemiology of any known circulating enterovirus types, non-polio enterovirus detection per se does not equate to clinical disease—some of the most prevalent non-polio enterovirus types detected in wastewater, such as species C coxsackieviruses (*i.e.,* CVA22 or CVA24),^10^ are very rarely identified in clinically diagnosed cases and currently not linked to any human disease. Changes in pathogenicity, such as documented for CVA6^28^, ^31^, ^32^ would remain invisible in the absence of clinical surveillance for HFMD and non-polio enterovirus -associated neurological disease.

In conclusion, this study documents a significant disease burden of enterovirus infections in the European Region, which is insufficiently characterized by the currently available surveillance data. We propose the implementation of broader standardised molecular screening, type identification and reporting of all enteroviruses, particularly from severely ill patients, such as those with AFP/AFM. This approach would enhance the quality of data generated by the Member States and improve our understanding of the burden of non-polio enterovirus infections without compromising poliovirus surveillance. Given the growing clinical relevance of non-polio enterovirus, the polio-free countries with low risk of polio importation but high routine immunization coverage could particularly benefit from implementation or expansion of AFM surveillance. In addition, we believe further efforts are needed to align the ENPEN surveillance data with the existing WHO reporting framework to more effectively monitor the disease activities of all enteroviruses in regions with different poliovirus immunization strategies.

## Contributors

HH, CKJ, KSMB and TKF conceptualised the study and drafted the manuscript. EVS, SH, and JEH provided the WHO data, advised on the study design and revised the drafts. HH and CK analysed the data. HH, CKJ, KSMB and TKF revised the drafts. All authors reviewed and approved the final manuscript. HH verified the data, and HH, CKJ and TKF had access to all raw data. HH had final responsibility for the decision to submit for publication.

## Data sharing statement

The data used in this study is the property of WHO Europe and will not be made publicly available in any format. Data may be made available for research purposes with inquiries directed to saxentoffe@who.int.

## Declaration of interests

HH and TKF are co-founders of ENPEN and declare conference and workshop attendance financed by The European Society of Clinical Microbiology and Infectious Diseases (ESCMID) and European Society of Clinical Virology (ESCV). CKJ and KSMB are members of ENPEN and declare conference and workshop attendance financed by European Society of Clinical Virology (ESCV). In addition, CKJ and TKF reports contracted work with WHO on polio- and non-polio surveillance. EVS, SH, and JEH are employees of the WHO Regional Office for Europe and have no conflicts of interest to declare.
