# Supplementary material for: Enterovirus circulation in the WHO European region, 2015–2022: a comparison of data from WHO's three core poliovirus surveillance systems and the European Non-Polio Enterovirus Network (ENPEN)
Source: Lancet Reg Health Eur. 2025 Apr 11;53:101292. doi: 10.1016/j.lanepe.2025.101292 (PMC12023770; doi:10.1016/j.lanepe.2025.101292)

**Supplementary Material**

**Supplementary Figure 1. Enterovirus types detected per year in each surveillance system.**

**
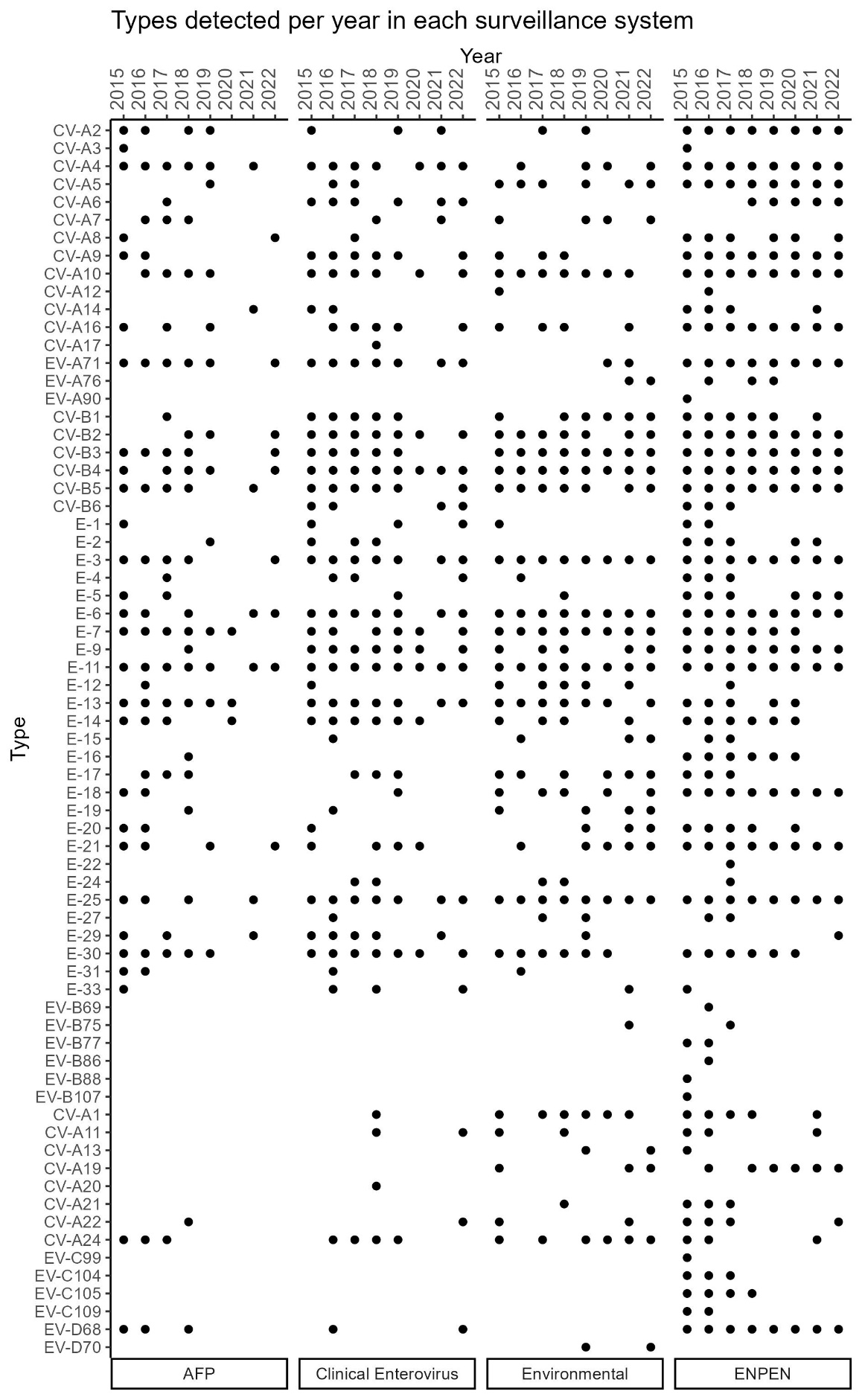
**

**Supplementary Figure 2.** Non-polio enteroviruses detected via AFP surveillance in Europe, 2015-2022 (n=105), over time (a) and by reporting country (b).

a)

b)
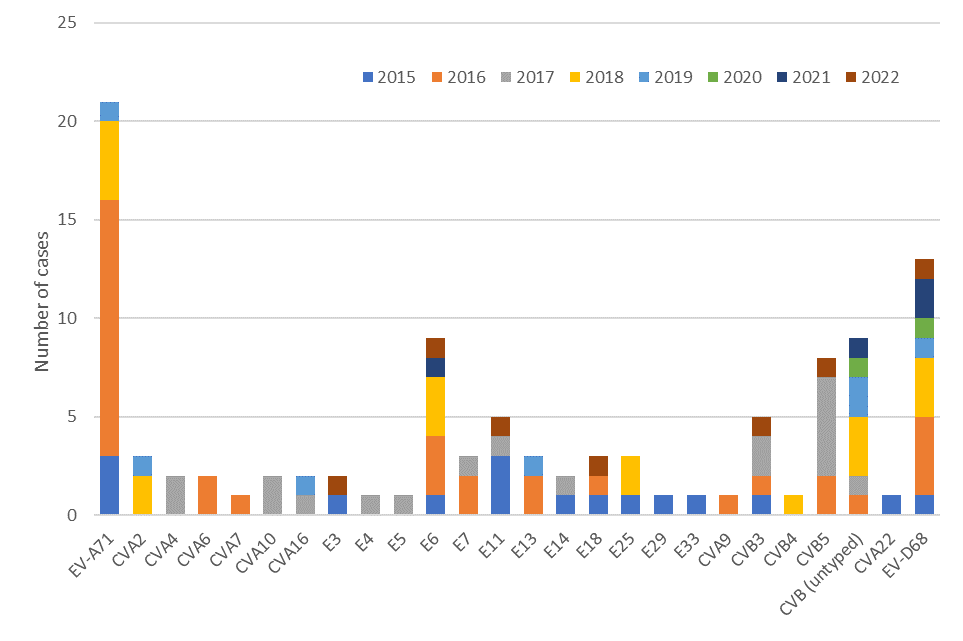

Supplement: Supplementary Material [file mmc1.docx]
